# Beneficial effects of short-term combination exercise training on diverse cognitive functions in healthy older people: study protocol for a randomized controlled trial

**DOI:** 10.1186/1745-6215-13-200

**Published:** 2012-10-29

**Authors:** Rui Nouchi, Yasuyuki Taki, Hikaru Takeuchi, Hiroshi Hashizume, Takayuki Nozawa, Atsushi Sekiguchi, Haruka Nouchi, Ryuta Kawashima

**Affiliations:** 1 Human and Social Response Research Division, International Research Institute of Disaster Science, Tohoku University, 4-1 Seiryo-machi, Sendai, 980-8575, Japan; 2 Smart Ageing International Research Centre, Institute of Development, Aging and Cancer, Tohoku University, 4-1 Seiryo-machi, Sendai, 980-8575, Japan; 3 Japanese Society for the Promotion of Science, 8 Ichibancho, Tokyo, 102-8472, Japan; 4 Department of Community Medical Supports, Tohoku Medical Megabank Organization, Tohoku University, 2-1 Seiryo-machi, Sendai, 980-8573, Japan; 5 Division of Developmental Cognitive Neuroscience, Institute of Development, Aging and Cancer, Tohoku University, 4-1 Seiryo-machi, Sendai, 980-8575, Japan; 6 Department of Functional Brain Imaging, Institute of Development, Aging and Cancer, Tohoku University, 4-1 Seiryo-machi, Sendai, 980-8575, Japan

## Abstract

**Background:**

Results of previous studies have shown that exercise training can improve cognitive functions in healthy older people. Some studies have demonstrated that long-term combination exercise training can facilitate memory function improvement better than either aerobic or strength exercise training alone. Nevertheless, it remains unclear whether short-term combination exercise training can improve diverse cognitive functions in healthy older people or not. We investigate the effects of four weeks of short-term combination exercise training on various cognitive functions (executive functions, episodic memory, short-term memory, working memory, attention, reading ability, and processing speed) of healthy older people.

**Methods:**

A single-blinded intervention with two parallel groups (combination exercise training; waiting list control) is used. Testers are blind to the study hypothesis and the participants’ group membership. Through an advertisement in a local newspaper, 64 healthy older adults are recruited and then assigned randomly to a combination exercise training group or a waiting list control group. Participants in the combination exercise training group must participate in the short-term combination exercise training (aerobic and strength exercise training) three days per week during the four weeks (12 workouts in total). The waiting list group does not participate in the combination exercise training. The primary outcome measure is the Stroop test score: a measure of executive function. Secondary outcome measures are assessments including the Verbal Fluency Task, Logical Memory, First and Second Names, Digit Span Forward, Digit span backward, Japanese Reading Test, Digit Cancellation Task, Digit Symbol Coding, and Symbol Search. We assess these outcome measures before and after the intervention.

**Discussion:**

This report is the first of a study that investigates the beneficial effects of short-term combination exercise training on diverse cognitive functions of older people. Our study is expected to provide sufficient evidence of short-term combination exercise’s effectiveness.

**Trial registration:**

This trial was registered in The University Hospital Medical Information Network Clinical Trials Registry (Number UMIN000007828).

## Background

Cognitive functions decline with age. For instance, older people might experience a decline in several cognitive functions such as memory [1], attention [2], executive functions [3,4], and processing speed [5]. Decline in cognitive ability engenders difficulty in performing basic daily living activities [6,7]. Consequently, maintaining or improving cognitive function in older adults is drawing increasing attention [8-15].

Previous studies have demonstrated that physical exercise training can improve cognitive functions in the healthy older people [14-20]. Physical exercise training can be of two types: aerobic exercise training and strength exercise training [18]. Aerobic exercise training is defined as structured exercise programs involving the use of large muscle groups for extended periods of time in activities that are rhythmic in nature, including but not limited to walking, stepping, running, swimming, cycling, and rowing [21]. Strength exercise training uses resistance against the force of muscular contraction to build strength, anaerobic endurance, and skeletal muscle mass. Strength exercise training often uses gravity to oppose muscle contraction [19,22,23]. Previous studies using randomized controlled trials (RCT) have revealed that aerobic exercise training alone and strength exercise training alone improved memory in healthy older people [22-24].

Some previous reports have described that combination exercise training, which combines exercise training of various types (for example, aerobic and strength exercise training) can also facilitate improvement of cognitive functions [20,21,25]. Williams and Lord [26] investigated whether combination exercise training for 42 weeks could improve cognitive functions in healthy adults or not. Results showed that the combination exercise training group improved processing speed and memory. Lautenschlager *et al*. [27] investigated the beneficial effects of 24 weeks of combination exercise training on the cognitive functions of older people with Alzheimer disease. The results showed that the combination exercise training group, which performed walking and strength exercise training, exhibited more significant improvement than a control group, which received educational classes, in the Alzheimer Disease Assessment Scale (ADAS) and word recall tests. Moreover, a meta-analysis showed that combination training had a larger effect than aerobic exercise training alone [14]. Consequently, combination exercise training appears to be the most effective exercise training for improving cognitive function.

### Purpose of this study

Although earlier studies showed that long-term (>24 weeks) combination exercise training can improve memory functions in elderly people [14,20,21,25,26], unresolved issues remain. First, it remains unclear whether or not short-term combination exercise training, for example, four weeks, can improve cognitive functions in healthy older people. Second, it is unclear whether the combination exercise training can improve diverse cognitive functions (for example, executive functions and attention) in healthy older people or not. Consequently, this study was conducted to investigate whether four weeks of combination exercise training can improve diverse cognitive functions in healthy older people or not. To reveal the beneficial effects of combination exercise training on widely various cognitive functions, we conduct a single-blinded RCT. Testers are blinded to the study hypothesis and the group membership of participants. We assess a broad range of cognitive functions. The measured cognitive functions are divisible into seven categories: executive functions, episodic memory, short-term memory, working memory, reading ability, attention, and processing speed.

## Method

### Randomized controlled trial design and setting of this trial

This study, which was registered in the University Hospital Medical Information Network (UMIN) Clinical Trial Registry (UMIN000007828), is an RCT conducted in Sendai city, Miyagi prefecture, Japan. Written informed consent to participate in the study will be obtained from each participant before enrollment. The protocol of this study and informed consent were approved by the Ethics Committee of the Tohoku University Graduate School of Medicine.

**Figure 1:**
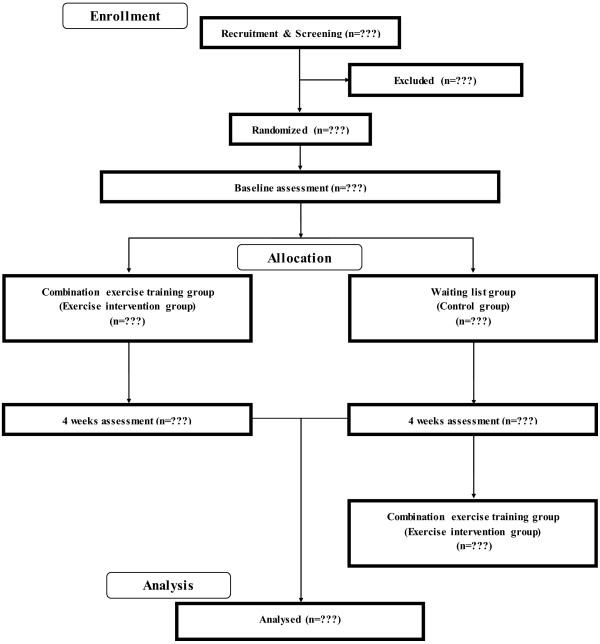
CONSORT flowchart.

To assess the impact of short-term combination exercise on diverse cognitive functions in healthy older people, we use a single-blinded intervention with two parallel groups: a combination exercise training group and a waiting list control group. Testers are blind to the study’s hypothesis and the group membership of participants. The Consolidated Standards of Reporting Trials (CONSORT) statement [28] (http://www.consort-statement.org/home/) was used as a framework for developing the study methodology (Additional file 1). The trial design is presented in Figure 1.

### Recruitment and selection of participants

Participants are recruited from the general population through advertisements in the local town paper and local newspaper. Interested participants are screened using a semi-structured telephone interview. After the telephone interview, participants are invited to visit Tohoku University for a more detailed screening assessment and to provide written informed consent.

### Inclusion and exclusion criteria

The purpose of this intervention is to investigate the beneficial effects of combination exercise training on diverse cognitive functions in healthy older adults. Criteria include participants who report themselves as right-handed, native Japanese speakers, unconcerned about their own memory functions, not using medications known to interfere with cognitive functions (including benzodiazepines, antidepressants, and other central nervous system agents), and having no disease known to affect the central nervous system, including thyroid disease, multiple sclerosis, Parkinson disease, stroke, severe hypertension (systolic blood pressure over 180, diastolic blood pressure over 110), and diabetes. The age of participants is more than 60 years old. We elicit the above information using a semi-structured telephone interview and self-report questionnaire. We check blood pressure at the start of this intervention study.

There are two reasons why we include only right-handed people. First, previous studies showed the association of handedness with cognitive functions for older people [29]. Second, we will investigate the effects of the combination exercise training on brain structures or brain functions using neuroimaging techniques such as magnetic resonance imaging after this intervention. In the neuroimaging studies, we usually use only right-handed people because of differences of lateralization of brain functions between right- and left-handed people. Language is more likely to be lateralized differently in left-handed people than in right-handed people. It means that in right-handed people, language functions such as grammar and vocabulary are most likely to be dependent on brain regions in the left hemisphere [30]. On the other hand, in left-handed people, it is more likely that left and right hemispheric brain regions will be involved [30,31]. To minimize the influence of subclinical degenerative conditions, the following exclusion criteria are employed. Criteria exclude participants who have an Intelligence Quotient (IQ) less than 85 derived from the Japanese reading test (JART) [32], a score of Mini Mental Status Examination (MMSE) less than 26 [33], and a score of Frontal Assessment Battery at bedside (FAB) less than 12 [34]. MMSE [33] is the most widely used screening instrument for the detection of cognitive impairment in older adults. MMSE is a 20-item instrument. The items of MMSE measure orientation for place and time, memory and attention, language skills, and visuospatial abilities. MMSE is scored from 0 to 30. Lower scores of MMSE indicate greater degrees of general cognitive dysfunction. The primary measure is the total score of this task (max = 30). FAB [34] evaluates executive functions. FAB consists of six subtests, namely, those for similarities (conceptualization), lexical fluency (mental flexibility), motor series (programming), conflicting instructions (sensitivity to interference), go/no-go (inhibitory control), and prehension behavior (environmental autonomy). FAB is scored from 0 to 18. Lower scores of FAB indicate greater degrees of executive dysfunction. The primary measure is the total score of this task (max = 18). This criterion is the same as our previous study, which investigated the beneficial effects of cognitive training using video games for four weeks [13]. Participants who participate in other cognitive-related intervention studies or other exercise intervention studies will be excluded.

### Randomization

Randomization is designed to take place after receiving the informed consent statement. A researcher (RN), who has no contact with the study participants, assigns participants to either the combination exercise group or the waiting list group by a random allocation sequence. The random allocation sequence will be generated by the Microsoft Excel 2003 program with the RAND function with no blocks and restrictions. Letters are used to inform participants of their allocation.

### Intervention group (combination exercise training group)

The intervention group receives the following combination exercise training, which was developed by Curves (Curves Japan Co., Ltd.). The combination exercise combines training of three types (aerobic, strength, and stretching; http://www.curves.co.jp/consistency/program/). Participants perform the combination exercise training three days per week throughout the four weeks (12 workouts in total). Each circuit-style workout consists of 12 strength training exercises (chest press/seated row, squat, shoulder press/lateral pull, leg extension/leg curl, abdominal crunch/back extension, lateral lift, elbow flexion/extension, horizontal leg press, pectoral deck, oblique, hip abductor/adductor, gluteus; http://www.curves.co.jp/consistency/program/point01/). The strength training machines contain calibrated pneumatic resistance pistons that allow for opposing muscle groups to be trained in a concentric-only fashion. Participants are informed of the proper use of all equipment and are instructed to complete as many repetitions in a 30-s time period. In a continuous, interval fashion, participants perform floor-based aerobic training (for example, running/skipping in place, and arm circles) on recovery pads for a 30-s time period after each resistance exercise in an effort to maintain a consistent exercise heart rate corresponding to 60 to 80% of their maximum heart rate. All workouts are supervised by trained exercise instructors who assist with proper exercise technique and maintenance of adequate exercise intensity. Participants must complete two circuits (24 min). After two rotations, participants do standardized whole-body stretching training (6 min). The whole-body stretching training consists of 12 stretching exercises (Achilles' tendon, sole of the foot, thigh, armpit, shoulder, shoulder/upper arm, chest/arm, shoulder/chest/arm, waist, back of knee, base of thigh, back; http://www.curves.co.jp/consistency/program/point02/).

### Waiting list group (no combination exercise training group)

The wait-listed group receives no intervention. Those participants are informed by letter that they are scheduled to receive an invitation to participate after a waiting period of four weeks. We ask the wait-list group not to go to the gym or not to join exercise programs during the waiting period. The previous study [35] suggested that the wait-listed group tends to show a resentful demoralization and to withdraw from the study. Based on this suggestion, 1) we set a short-term waiting duration (four weeks) and 2) to keep the motivation to participate in this study, we will send a short letter to the wait-list group at the midpoint of the waiting duration. In this study, we use no active control group such as a stretch exercise training group alone or placebo group such as a social contact group because results of previous intervention reports describe that no difference exists in cognitive or functional improvement between the stretch exercise training group (active control group) and non-exercise control group (control group) [36] or between the social-contact group (placebo group) and no-social-contact groups (control group) [37,38]. Moreover, using the wait-list control group has the advantage of letting everyone in the study receive the new intervention such as the combination exercise training (sooner or later). Thus, we use the wait-list control group in this intervention study.

### Overview of cognitive function measures

To evaluate the beneficial effects of combination exercise on cognitive functions, we assess a broad range of cognitive functions (Table 1). Measures of the cognitive functions are divisible into seven categories (executive functions, episodic memory, short-term memory, working memory, reading ability, attention, and processing speed). Executive functions are measured using the Stroop Test (ST) [39] and Verbal Fluency Task (VFT) [40]. Episodic memory is measured using Logical Memory (LM) [41] and First and Second Names (FS-N) [42]. Short-term memory is measured using Digit Span Forward (DS-F) [43]. Working memory is measured using Digit Span Backward (DS-B) [43]. Reading ability is measured using the Japanese Reading Test (JART) [32]. Attention is measured using the Digit Cancellation Task (D-CAT) [44]. Processing speed is measured using Digit Symbol Coding (Cd) [43] and Symbol Search (SS) [43]. Details of all tasks are described below.

**Table 1:** Summary of cognitive function measures

**Cognitive function**	**Task**
Executive functions	Stroop test
	Verbal fluency task
Episodic memory	Logical memory
	First and second names
Short-term memory	Digit span forward
Working memory	Digit span backward
Reading ability	Japanese reading test
Attention	Digit cancellation task
Processing speed	Digit symbol coding
	Symbol search

We assess these cognitive function measures before and after the intervention period (four weeks). The primary outcome measure is ST, which we selected because 1) previous studies using exercise training showed that exercise training can improve executive functions [45,46], 2) ST is a task that is often used to measure executive functions [47,48], and 3) ST has been standardized, with high reliability and validity in Japanese populations [39,49].

***ST***: Stroop Test (ST) measures executive function including response inhibition and impulsivity. Hakoda's version is a paper and pencil version ST [39]. In this test, participants must check whether their chosen answers are correct, unlike the traditional oral-naming ST. We use a reverse ST and an ST. In the reverse ST, in the leftmost of six columns, a word naming a color is printed in another color (for example, ‘red’ is printed in blue letters); each of the other five columns is filled with five different colors from which participants must check the column whose color matches the written word in the leftmost column. In the ST, in the leftmost of six columns, a word naming a color is printed in another color (for example, ‘red’ is printed in blue letters) and the other five columns contain words naming colors. Participants must check the column containing the word naming the color of the word in the leftmost column. In each task, participants are instructed to complete as many of these exercises as possible in 1 min. The primary measure for this task is the number of correct items.

***VFT***: Verbal Fluency Task (VFT) measures executive function. We use the Japanese version of VFT [40], which has two tasks (the Letter Fluency Task (LFT) and Category Fluency Task (CFT)). In LFT, a Japanese letter, ‘ka’, is given to each participant, who is then asked to generate common nouns beginning with this letter - as many as possible in 60 s. In CFT, a category name (Animal) is given to each participant, who is then asked to generate many words of a certain category (Animal). The participants are instructed not to include proper nouns or to repeat one that has already been stated. The primary measure for this task is the number of words reported. The reliability and validity of Japanese LFT were demonstrated by Ito [40].

***LM***: Logical Memory (LM) evaluates the performance of episodic memory. LM is a subtest of the Wechsler Memory Scale-revised (WMS-R) [41]. LM consists of two short, paragraph-length stories (Story A and Story B). In LM, participants must memorize the short story. The stories are scored in terms of the number of story units recalled, as specified in the WMS-R scoring protocol. We use either Story A or Story B. The primary measure for this task is the number of correct story units recalled.

***FSN***: First and Second Names (FSN) evaluates memory ability in everyday life. FSN is a subset of Rivermead Behavioural Memory Test (RBMT) [42]. RBMT measures episodic memory as it is used in everyday life. Therefore, subsets of RBMT are similar to everyday situations. In FSN, participants must memorize first and second names with faces (photographs). Subsequently, they must recall the first and the second names when the face is shown again later. We use four faces (four first names and four second names). The primary measure of this test is the total number of correct answers in both first and second names. The maximum raw score of FSN is 8.

***DS*:** Digit Span (DS) is a subtest in Wechsler Adult Intelligence Scale-third edition (WAIS-III) [43]. Digit Span, which has two subsections (DS-F and DS-B), evaluates short-term memory and working memory. DS-F measures short-term memory simply by requiring participants to repeat numbers. DS-B measures working memory by requiring participants to memorize numbers and to repeat the numbers in inverse order. For DS-F, participants repeat numbers in the same order as they were read aloud by the examiner. For DS-B, participants repeat numbers in the reverse order of that presented aloud by the examiner. In both, the examiner reads a series of number sequences which the examinee must repeat in either forward or reverse order. DS-F has 16 sequences. DS-B has 14 sequences. The primary measures of this test are raw scores that reflect the number of correctly repeated sequences until the discontinue criterion (that is, failure to reproduce two sequences of equal length) is met [43]. The maximum raw score of DS-F is 16. The maximum raw score of DS-B is 14.

***JART*:** The Japanese Reading Test (JART) measures reading ability [32]. JART is a Japanese version of the National Adult Reading Test (NART), which has a reading test of 50 irregularly spelled words in English (for example, ache) [50]. JART is a reading test comprising 25 Kanji compound words (for example, 親父, 煙草). The reading stimuli are printed out randomly for reading. The participants are asked to read each Kanji compound word aloud. This task assesses reading ability and IQ. The primary measure for this task is the number of correct items.

***D-CAT*:** Digit Cancellation Task **(**D-CAT) evaluates attention [44]. The test sheet consists of 12 rows of 50 digits. Each row contains five sets of numbers 0 to 9 arranged in random order. Consequently, any one digit appears five times in each row with randomly determined neighbors. D-CAT comprises three such sheets. Participants are instructed to search for the target number(s) that had been specified to them and to delete each one with a slash mark as quickly and as accurately as possible until the experimenter sends a stop signal. Three trials exist, first with a single target number (6), second with two target numbers (9 and 4), and third with three (8, 3, and 7). Each trial is given for 1 min. Consequently, the total time required for D-CAT is 3 min. In the second and third trials, it is emphasized that all the target numbers instructed should be cancelled without omission. The primary measure of this test is the number of hits (correct answers). We use only the number of hits in the first trial.

***Cd*:** Digit Symbol Coding (Cd) is a subtest of WAIS-III [43]. This test measures processing speed. For Cd, the participants are shown a series of symbols that are paired with numbers. Using a key within a 2 min time limit, participants draw each symbol under its corresponding number. The primary measure of this test is the number of correct answers.

***SS*:** Symbol Search (SS), a subtest of WAIS-III containing 60 items [43], measures processing speed. For this subtest, participants visually scan two groups of symbols (a target group and a search group) and report whether either of the target symbols matches any symbol in the search group. Participants respond to as many items as possible within a 2 min time limit. The primary measure of this test is the number of correct answers.

### Psychological questionnaire

To evaluate the beneficial effects of combination exercise on satisfaction and quality of life (QOL) for participants, we use some questionnaires. We ask participants to answer the questionnaires related to the subjective feelings (1; motivation of continuing the combination exercise during the intervention period, 2; fatigue during the intervention period, 3; satisfaction of the intervention during the intervention period, 4; enjoyment of the combination exercise during the intervention period) after the intervention period. Participants rate these questionnaires using a nine-point scale (for motivation scale, from 1 = very low to 9 = very high; for fatigue scale, from 1 = very low to 9 = very high; for satisfaction scale, from 1 = very low to 9 = very high; for enjoyment scale, from 1 = very low to 9 = very high). To evaluate QOL, we use Japanese versions of WHOQOL-BREF (WHO/QOL-26) [51]. The QOL-26 is a short version of the WHOQOL and is a self-rating instrument that assesses individuals’ perceptions of their position in life in the context of the culture and value system in which they live and in relation to their goals, expectations, standards, and concerns. The QOL-26 has 26 items with 5 subscales: Physical (physical state), Psychological (cognitive and affective state), Social (interpersonal relationships and social roles in life), Environmental (relationships to salient features of the environment), and Global (meaning in life, or overarching personal beliefs). A 5-point response category is used, ranging from 1, which indicates ‘strongly disagree’, to 5, which indicates ‘strongly agree’. All responses on each subscale are added within each subscale. The total score is the sum of the first 5 subscales. The QOL of the respondent is evaluated using the scores of the first 5 subscales and their total score. A higher score indicates better QOL.

### Sample size

Our sample size estimation is based on the change score in the reverse Stroop task, which is the primary outcome in this study. This study uses eta squared (η^*2*^) as an index of effect size. As a descriptive index of strength of association between an experimental factor (main effect or interaction effect) and a dependent variable, η^*2*^ is defined as the proportion of total variation attributable to the factor, and it ranges in value from 0 to 1 [52]. Using information (the sums of squares for total; SS total, the sum of squares for factor; SS factor) reported in an analysis of covariance (ANCOVA) summary table, we calculate η^*2*^ (SS factor divided by SS total). SS factor is the variation attributable to the factor and SS total is the total variation, which includes the SS factor and the sum of squares for error. In actuality, η^*2*^ ≥0.01 is regarded as a small effect, η^2^ ≥0.06 as a medium effect, and η^*2*^ ≥0.14 as a large effect [52]. We expect to detect a large effect size (η^2^ = 0.14) of the change score (post-training score minus pre-training score) in the reverse Stroop task between combination exercise and waiting list groups. The sample size was determined using G*power [53,54] based on 80% power, a two-sided hypothesis test, an alpha level of 5%, an ANCOVA model that includes a baseline reverse Stroop task score, age and sex as a covariate. The sample size calculation indicated that we need 32 participants in each of the combination exercise and waiting list groups with consideration of a 20% drop-out rate.

### Analysis

This study is designed to evaluate the beneficial effect of the short-term combination exercise in older people. We calculate the change score (post-training score minus pre-training score) in all cognitive function measures. We conduct an ANCOVA for the change scores in each cognitive test. The change scores are the dependent variable. Groups (combination exercise, waiting list) are the independent variable. Pre-training scores in the dependent variable, sex, age categories are covariates to adjust for background characteristics and to exclude the possibility that any pre-existing difference of measure between groups affects the result of each measure. The level of significance is set as *P* <0.05. This study reports η^*2*^ as an index of effect size (please see the detail in Sample Size). Missing data are imputed using Missing Value Analysis in the Statistical Package for the Social Sciences (SPSS). In particular, we impute missing values using maximum likelihood estimation based on the expectation-maximization algorism with the observed data in an iterative process [55]. All randomized participants are included in the analyses in line with their allocation, irrespective of how many sessions they complete (intention-to-treat principle). All analyses are performed using the SPSS (version 18 or higher; SPSS Japan Inc.).

## Discussion

This study is designed to investigate the beneficial effects of short-term combination exercise training on diverse cognitive functions such as executive functions, episodic memory, short-term memory, working memory, reading ability, attention, and processing speed in healthy older people.

This study has several strengths compared to earlier studies using exercise training for older people. First, we use hydraulic exercise machines to increase muscle strength. The hydraulic machines present some advantages. 1) The harder participants push or pull and the faster they move, the greater the resistance they create. Consequently, participants can adjust the strength exercise training load. 2) Hydraulic exercise machines are designed to be used by all muscle groups. Machines exist to work the upper body, lower body, abdominals, and obliques. Therefore, participants can easily develop muscles throughout the body. 3) The hydraulic exercise machines are safer for older people to use than exercise machines of other types because the loads of the hydraulic machines disappear when participants stop moving such as pushing or pulling.

Second, training periods (30 min per day, three days a week for four weeks) in our combination exercise training are shorter than those in previous exercise training studies. Meta-analysis showed that earlier exercise training studies used long-term training periods (>24 weeks) [14]. Considering reduced costs for older people, shorter intervention studies using exercise training are also needed. Consequently, our short-term combination exercise training can offer important suggestions for methods used in the exercise training field.

Third, some previous studies showed that combination exercise training improved certain cognitive functions such as memory functions [26,27]. However, it remains unclear whether or not combination exercise training can improve cognitive functions of other types. In this study, we use diverse cognitive function measures simultaneously. Therefore, we can investigate the beneficial effects of the short-term combination exercise training on diverse cognitive functions such as executive functions, episodic memory, and processing speed.

This study investigates the beneficial effects of short-term combination exercise training on the cognitive functions in older people. If findings indicate that our short-term combination exercise training is very effective, then we will conduct future studies. For instance, assuming the validity of effects of short-term combination exercise training on diverse cognitive functions, we can conduct the same RCT for non-healthy older people such as those with dementia or depression. Additionally, we will investigate the beneficial effects of our combination exercise training on brain functions and brain structures using neuroimaging techniques such as magnetic resonance imaging.

We discuss the risks of this study. As there are no known side effects and there have not been any reports of adverse reactions to the combination exercise program for older people, we do not anticipate that any participants will be exposed to any unnecessary risks. However, there are risks of using the strength training machines. To reduce these risks, we conduct the combination exercise training with the presence of professional trainers, who have all been approved for the study by the Ethics Committee and have worked for more than two years as a trainer using the combination exercise training.

There are some limitations in this study. First, the intervention period of this study is shorter than that of the previous studies. The short-term intervention such as four weeks is one of advantages or strong points of this study. The previous study for older people demonstrated that four weeks’ intervention period could be enough to improve some cognitive functions (the main purpose of this study) [13]. However, it may not be long enough for older people to gain cardiovascular health, to control weights and to get into the habit of exercise. Gaining cardiovascular health or controlling weight is not important for our participants because we recruit healthy older people who are not suffering from obesity and hypertension. The habit of exercise would keep cognitive functions and mental health in later life. In future study, it will be necessary to investigate whether or not the short-term intervention would facilitate older people gaining cardiovascular health or getting into the habit of exercise.

Second, we do not use an active control group who participates in other types of exercise programs. The reasons why we use the wait-list group are that 1) previous studies showed no difference exists in cognitive or functional improvement between the stretch exercise training group (active control group) and non-exercise control group (control group) [36] and 2) using the wait-list group has the advantage that all participants can experience the combination exercise training. From a study design point of view, it would be better than using the active control group. This is the first study to demonstrate the beneficial effects of the combination exercise training on the cognitive functions. If we find the combination exercise training to be effective for older people, we should conduct new intervention studies that compare the combination exercise training to other exercise training or other cognitive training.

We should discuss the generalizability of this study because this study is a single center study (in Sendai city, Japan) using very healthy older people. The protocol of the combination exercise training uses simple hydraulic exercise machines and does not depend on language ability or Japanese language. Thus, we assume that the combination exercise can be applied to other healthy older people in Japan and would be available to everyone around the world. As we mentioned before, there are no known side effects and there have not been any reports of adverse reactions to the combination exercise program. If people who have low IQ, any CNS disease or cognitive impairment participate in the combination exercise training, the chance of an accident may increase. In that case, we should conduct the combination exercise training with expert help (for example, a medical doctor, or psychologist).

It is important to discuss the possibility of the ceiling effect in cognitive measures. Because we will recruit very healthy older people (please see the Inclusion and Exclusion Criteria section), there would be a possibility that the scores of cognitive functions in participants before the intervention are close to the maximum or the highest scores. Thus, the short-term intervention would not lead to improvement in cognitive functions of the intervention group compared to that of the control group. However, we measure a wide range of cognitive functions and use standardized cognitive measures. Moreover, our previous intervention study using video games for healthy older people [13] showed improvement of cognitive function after the four weeks’ cognitive intervention. For that reason, we would reduce the possibility of the ceiling effect in this study.

In summary, this report is the first of a study assessing the beneficial effects of short-term combination exercise on diverse cognitive functions in older people. Our study is designed to provide sufficient evidence of the effectiveness of short-term combination exercise. Given that most cognitive functions decrease with age [56] and that these functions are correlated strongly with daily life activities [6,7,57], our results can elucidate the effects of exercise training for older people.

## Trial status

Recruitment of participants, beginning in May 2012, is expected to end in January 2013. Patient recruitment is ongoing.

## Abbreviations

ADAS: Alzheimer Disease Assessment Scale; ANCOVA: Analysis of covariance; CFT: Category Fluency Task; Cd: Digit symbol coding; CONSORT: Consolidated Standards of Reporting Trials; D-CAT: Digit cancellation task; DS-B: Digit span backward; DS-F: Digit span forward; FAB: Frontal Assessment Battery; FS-N: First and second names; IQ: Intelligence Quotient; JART: Japanese reading test; LFT: Letter Fluency Task; LM: Logical Memory; MMSE: Mini Mental Status Examination; QOL: Quality of life; RCT: Randomized controlled trial; RBMT: Rivermead Behavioural Memory Test; SPSS: Statistical Package for the Social Sciences; SS: Symbol Search; ST: Stroop Test; UMIN: University Hospital Medical Information Network; VFT: Verbal fluency task; WAIS-III: Wechsler Adult Intelligence Scale-third edition; WMS-R: Wechsler Memory Scale-revised.

## Competing interests

All authors declare they have no competing interests.

## Authors’ contributions

RN designed, developed the study protocol and calculated the sample size. RN and HN searched the literature, selected cognitive function measures, created manuals to conduct and rate cognitive measures, and recruited testers for cognitive function measures. HN conducted cognitive function measures and rated these cognitive function measures with testers. RN supervised testers. RN wrote the manuscript with YT, HT, HH, YN, AS, HN, and RK Additionally, RK provided advice related to the study protocol. All authors read and approved the final manuscript.

## Supplementary Material

Additional file 1 CONSORT 2010 checklist of information to include when reporting a randomized trial. Click here for file
